# Interprofessional practice: beyond competence

**DOI:** 10.1007/s10459-019-09879-4

**Published:** 2019-03-02

**Authors:** Brenda Flood, Liz Smythe, Clare Hocking, Marion Jones

**Affiliations:** 1 Faculty of Health and Environmental Sciences, Auckland University of Technology, Auckland, New Zealand; 2 Graduate Research School, Auckland University of Technology, Auckland, New Zealand

**Keywords:** Competency, Interprofessional practice, Phenomenology, Health professional education

## Abstract

Interprofessional practice is commonly discussed in the literature in terms of competencies. In this study we move away from the theoretical notions of criteria, concepts and guidelines to adopt an ontological approach which seeks to stay as close to the lived experience as possible. Our research asked 12 participants from a variety of health disciplines to tell their stories of working interprofessionally. We sought to glean meaning from the lived experience. Our phenomenological hermeneutic approach and interpretation were informed by Heidegger and Gadamer. Rather than offering a thematic overview, in this article we share three stories from the research that were congruent with other stories. The first, told by a doctor, is of a resuscitation in an emergency department. It shows how the effective working together of the interprofessional team was more than each member following a resuscitation protocol. There was ‘something’ about how they worked together that made this story stand out, even though the patient died. The second story showcases how ‘who’ the person is makes a difference. This nurse makes an effort to get to know other staff as people, to find common interests. In such a way interprofessional practice comes to flourish. The third story shows how a physiotherapist and a psychologist joined in conversation to seek innovative possibilities for a challenging situation. In such a way each built on the others expertise and were excited at the success they achieved for the patient. From these ontological accounts we have come to see that interprofessional practice flourishes when practitioners are their authentic, caring selves. Who the person is matters.

## Introduction

Competencies are so pervasive in health professional education and practice that they have been dubbed one of the field’s “most cherished ideas” (Lingard 2009, p. 625), an idea taken up as the cornerstone of interprofessional practice. By nature, educational institutions and health care providers seek ways to set standards for achievement and performance, and then determine ways to measure that those pre-determined standards are met. In so doing, educators and health care providers have followed established scientific practice of objectifying things in order to gain knowledge of them (Harman 2007). While international agreement on the definition of competence has not been achieved, there is broad agreement that competencies are skills and knowledge that meld together into directly observable abilities. Competencies thus help to define what learners need to master and, because they are observable, render learners’ progress towards achievement of competence measurable (Kumagai 2014). There is an assumption that as the factors contributing to specific competencies become clearer, the criteria against which to evaluate the achievement of competence will become more precise (Nagelsmith 1995; Reeves et al. 2009). The standard and the measure both relate to the concept of ordering ‘competence’. It is akin to a warrant of fitness. Once the warrant is granted, it is expected the person will maintain function at the desired level. All is under control. The pre-planning assumes the ‘warranted’ people are practising in the manner prescribed. Everyone is satisfied, or are they?

There have been critical voices. Competencies have been decried as a “minimalistic discourse” (Talbot 2004, p. 587) originating in American industry that fractures competent performance into “basic unitary skills” (Fernandez et al. 2012, p. 363). That fracturing, irrespective of its intent to make human performance intelligible, can be viewed as objectifying knowledge in ways that “de-live” the human experience, rendering it an oversimplification or caricature with inevitable distortions (Harman 2007). Alert to that critique, definitions more encompassing of the complexities of expert professional practice incorporate “higher order competence” (Talbot 2004, p. 587) including judgement, values, emotions, and personal or character attributes (Fernandez et al. 2012; Talbot 2004). Reflecting concerns about whether it is possible to acquire or teach those attributes (Fernandez et al. 2012; Kumagai 2014), recent research has investigated whether already possessing traits such as emotional intelligence and learner autonomy hastens the process of acquiring competence (Park et al. 2015).

Alongside ongoing efforts to clarify the nature of competencies themselves, the field of interprofessional practice has established itself through the articulation of models of competency (Brewer and Jones 2013; Canadian Interprofessional Health Collaborative 2010; Interprofessional Education Collaborative 2016) and education and practice frameworks (Charles et al. 2010; Institute of Medicine 2015; World Health Organization 2010). Such scaffolding is essential to begin to build the ‘thing’, the curriculum, the graduate profile, the standards and expectations and the organisation of care. It is a taken for granted ‘truth’ that collaboration between members of diverse health professions can be broken down into competencies. One rendition of the scope of those competencies itemised the ability to discharge one’s role competently, appreciate the responsibilities and boundaries of one’s own and others’ roles, and collaborate on all aspects of care delivery and ongoing service review and improvement. Also included were teaching and learning from other disciplines, and being tolerant of the anticipated “misunderstandings, ambiguities, shortcomings and unilateral change in other professions” (Barr 1998, p. 185). Recent publications across the UK, Canada, North America and Australasia reflect broad agreement on the core constructs: communication, collaboration, teamwork and patient-centred care. While there is regional variability in the details, the perception that competencies are the foundation for education for interprofessional practice and workforce development seem ubiquitous (Bainbridge et al. 2010; Gunaldo et al. 2017; Reeves 2012; Thistlethwaite et al. 2014).

Our argument pointing to the limits of ‘competencies’ takes an ontological approach informed by Heidegger [1889–1976]. He challenges the notion of objectively describing and measuring human engagement. Such a method falls within the ontic approach of modern science, where the unstated aim is “to secure the calculability of nature” (2001, p. 105), in other words to plan with certainty and confidence that the outcomes of enactment will be as predicted. Within this kind of method, Heidegger (2001) argues: “thinking gets passed off as nothing more than a manipulation of operational concepts, representational models and models of thinking” (p. 107). As Heidegger reminds us (1971a), the essence of the ‘thing’ is not to be found in the scaffold, but in the playing-out. He gives the example of a jug. While we might assume the ‘thing’ to be the jug, what matters about the jug is what is poured into it as a holding vessel, and later poured out. In other words, how the jug goes about being a jug. In the ontic approach, little attention is given to how a jug is ‘experienced’, yet that is what matters.

## Aim and purpose

Our aim in this paper is to trouble the idea that competencies are a sufficient basis for the design of educational programmes and evaluation of competent practice. The study from which this discussion draws sought to return to the lived experience of interprofessional practice, to listen to stories of health practitioners involved in working with people from other disciplines. Our aim was to reveal what it means to ‘be’ and ‘become’ an interprofessional practitioner. The study took a hermeneutic phenomenological approach informed by Martin Heidegger [1889–1976] and Hans-Georg Gadamer [1900–2002]. Bringing a phenomenological approach to the lived experience of educational encounters and healthcare delivery is an established methodology (Giles et al. 2012; Glover and Philbin 2017; Landrum et al. 2017; Rocha 2016; Thomson 2016). Approval for this study was gained from the Auckland University of Technology Ethics Committee in October 2014.

## Methodology

It is important to lay out the philosophical assumptions of the move to an ontological understanding. Heidegger is a key philosopher in returning to discern the insights that arise from our experience of ‘being’. Sheehan (2014), in asking “What, after all, was Heidegger about?” talks of how in ‘being-there’ we may be thrown into an open space where we can begin to discern the meaningfulness of our experience, and the significance to ‘me’. However, in the everydayness we can all too easily be distracted by the voice of ‘They’ dictating how things are expected to be (Heidegger 1962). In such a manner we go about in a non-thinking mode (Heidegger 1968). Thomson (2016), drawing on Heidegger, points to needing to own our own experiences, yet recognises it is a “continual struggle… to become who we are” (p. 849). He talks of the choice to “*impose* pre-established standards” or to take on the work to “*disclose* and develop students’ distinctive path to a meaningful life” (p. 853, emphasis original). To *impose* is to stay bound to ontic pre-determined thinking. To *disclose* is to be in the open space where meaningfulness of ‘here and now’ is free to emerge. The interviews within this research sought to take participants to the open space, to invite them to reflect on their own experiences of practicing with colleagues from other disciplines, to enable the meaningfulness to emerge. The interpretive nature of our analysis embraces those hints of meaningfulness and seeks to dwell on them further.

## Method

This study was undertaken by the lead author/researcher (Flood 2017), in collaboration with the named co-authors/supervisors. Flood brought her own experiences from practice. When she told stories of how she worked interprofessionally with specific people in specific settings, she became excited. When she recounted the writing on ‘competency’ her enthusiasm waned. The supervisors brought long experience of phenomenological hermeneutic research that sought to discern meaning from lived experience. Smythe (2003), in exploring the notion of ‘safety’, challenged the concept that ‘competency’ in itself could make a situation ‘safe’. Rather she saw that “within the pressing, hemming world of practice” concepts, such as competency were translated into “lived reality, abounding with possibilities” (Smythe 2003, p. 202). Hocking and Smythe supervised, among others, a study that explored core concepts of occupational therapy (Reed et al. 2011), where the initial impetus had been the lack of attention to meaning that emerges from lived experience. Jones et al. (2014) in contrast was very familiar with helping establish the competencies for interprofessional practice. As an interprofessional team of 2 occupational therapists, a midwife and a nurse we lived the experience of working across disciplines, keeping each other open to insights that were different from our own personal experience.

Data collection involved in-depth, semi-structured interviews with 12 health professionals from nursing, occupational therapy, physiotherapy, speech and language therapy, medicine, social work, and midwifery. The interviews, which were recorded and transcribed, used a conversational style to gather participants’ understandings and perspectives of interprofessional practice. Heeding Heidegger’s (1962) call to seek to get as close to the primordial experience as possible meant trying to invite stories of ‘what happened’ rather than asking questions that asked for opinions. Immersion in the transcripts allowed stories and unifying themes of experience and meaning to emerge, many announcing themselves as important. Once a potential story was identified, it was recrafted into a logical and chronological order, careful to use the participants’ own words, editing grammar and punctuation where necessary and removing distracting details (Crowther et al. 2017). This process immerses the researcher in thinking about what has been said and aims to offer the ‘story’ in a compelling manner to enable reader to ‘see’ (van Manen 1990). Flood worked with the data in this way. The supervisory team engaged in discussing the emerging meanings. The crafted stories were returned to the participants to enable their approval. The intent was to bring their meaning to light, not to change the meaning (Smythe and Spence 1999). In the recrafting of this ‘telling’ by participants into stories, the authors were careful to identify those which conveyed something of the meaning of the phenomenon of interprofessional practice.

Interpretation focused on accounts strongly linked to ways of ‘being’ interprofessional. It is in the reading and recrafting that interpretation begins, helping to stay connected to the meanings that emerged from the text, bringing more depth and clarity to the interpretation process (Caelli 2001). In dwelling with, and gaining a deeper understanding of, the nature of events as experienced in everyday practice, a more thoughtful approach to the development of interprofessional learning was opened up, where the ‘being’ and ‘becoming’ can be foregrounded. van Manen (2014, p. 20) in talking about hermeneutic phenomenology states:

Phenomenological writing is not just a process of writing up or writing down the results of a research project. To write is to reflect; to write is to research. And in writing we may deepen and change ourselves in ways we cannot predict.

The interpretive process of writing and re-writing was accompanied by reading related extracts from Heidegger (who encourages a return to being in its everydayness) and Gadamer (who awakens us to the challenge of the interpretive process) (Schmidt 2006). Thus, the interplay between the philosophical notions and the insights that were emerging was constantly at play. An overview of the findings is reported elsewhere (Flood et al. 2019).

This article focuses on one insight arising from the study, that working in the ‘spirit’ of interprofessional practice goes beyond competencies. Interprofessional practice cannot be reduced to separate aspects, it is about everything. The things that call us, the spirit with which we engage in the phenomenon that is interprofessional practice and the things that safeguard and preserve interprofessional practice are all dependent on the other. Everything comes into play. There is a ‘spirit of interprofessional practice’ that is ‘known’ but beyond measure; ‘seen’ but beyond planning; ‘felt’ but beyond reduction to a skill. While recognising the predominance of the discourse around ‘competence’ in the interprofessional literature, our argument is that while such research and scholarship has been vital to understanding the tenets of interprofessional practice, in itself ‘competencies’ are not sufficient.

## Presentation of findings

The three stories that follow demonstrate the role and value that competencies play in healthcare contexts and also how practising in a spirit of interprofessional practice goes beyond what competencies can encompass. The stories show the emergent and allusive nature of interprofessional practice and reveal intangible qualities that sustain a way of being in such territory. Any number of stories from the research could have been selected to reveal aspects of what it means to ‘be’ interprofessional. The three shared here, resonated with the authors. There was an ‘inclining toward’ these texts, a feeling, a knowing, that they would best illustrate the argument put forward in this paper (Smythe and Spence 2012). They each show a unique aspect of the phenomenon. All identifying information has been removed from the stories and names replaced with pseudonyms.

Amanda, a senior emergency department doctor, describes interprofessional practice during a resuscitation.

I work in an emergency department and a particular interprofessional team that springs to mind is the resuscitation team who were called to a daytime resuscitation. The patient had an arrhythmia and arrived with the paramedics. There were also emergency medicine doctors, nurses, the medical registrar, a cardiologist, an orderly and an health care assistant (HCA) as well. There is a very clear prescribed algorithm to follow for a person with arrhythmia. Having a common goal, or a common mental model, absolutely brings the team to the same space that can pull everything together. Everyone knew what they were doing and had an awareness of what other people were doing. Despite the outcome for the patient… the patient died, I think they did have the best opportunity, if there was any opportunity to have survived, the patient would have. I still think it really was a successful resuscitation. We felt like it all worked really well and people worked well together, and we gave the patient the very best chance regardless of this particular outcome.

Those working together to resuscitate the patient in the emergency department on this day were following a pre-determined plan incorporating the competencies expected of a resuscitation team. They had prescribed roles and were following a clear process. Each would have been deemed to be able to competently provide high quality care by their employer. Perhaps what is striking about this story is that is has been singled out and told as a memorable experience. On this day, in this moment, the team worked well together. They had given the patient the best possible chance of survival. Not said, but implied is that on another day at another time things may not have worked so well. Something about the way the team worked on this day meant it was judged by a senior member of the team as “a really successful resuscitation”. As Amanda thought back, she recognised that each person worked really well, but also people worked well together. There is a ‘people’ factor here that cannot be known in advance. In the coming and going of a busy unit, it is rare for the same group of people to work together. It may be that colleagues do not always have foreknowledge of another person’s competence-under-stress. Certainly, they do not know how the synergy of this particular grouping of people will play out. Will there be respect for the leader’s judgement? Will each trust the other is performing as expected? Do they read the mood, pick up the non-verbals, respond in an attuned manner?

Heidegger says: “method holds all the coercive power of knowledge” (1971b, p. 74). The method of a team resuscitating a patient is about following the rules dictated by knowledge. It seems, however, that this team went beyond the rules. They were united in a spirit of doing their very best, thinking through this challenging situation where the skills they were employing were not having the desired effect. “To think is before all else to listen, to let ourselves be told something” (1971b, p. 76). There is a sense of openness in this story. Each person was open to what was going on, what was happening and not happening, what the situation was telling them. While protocol was no doubt guiding them, in itself protocol is not sufficient to achieve the harmony and synergy of a cohesive team. Heidegger talks of the ‘ringing of stillness’ as being the language of being. One can imagine the moment when this resuscitation was called to a halt and the patient declared ‘dead’. One can feel the long moment of sorrowful stillness. Yet, within this stillness is the knowing that something special had been enacted. The spirit of interprofessional practice had flourished. The experience was so much more than a demonstration of competence.^1^

Ricardo, a nurse working in an inpatient mental health facility tells a story about the value of relationships and getting to know others he works with.

I suppose with any other relationship with new people it’s kind of always finding some common ground to build on. I endeavour that when there’s a new colleague or new staff, I am just more friendly and have that relationship so at the end of it, when there’s a nice working relationship it makes working a lot more fun, a lot more easy and you burn out less. We have a new social worker who’s Japanese actually. I found that one of the nurses would make comments to me asking if she is friendly, because they notice she doesn’t really talk to people, she just goes there and talks with the patients and then leaves the nursing station. In fact that’s a different perception to me. I find her quite warm, I find her quite accommodating…. For me she’s always just an email away and I can always collaborate, but some colleagues find her a bit off maybe. I think it’s because for me I know a little Japanese so when I first met her I said ‘oh hey Konichiwa, blah, blah, blah’!! So there was already that sense of ‘hey we have something in common’. For me it’s not necessarily because she’s a social worker and I’m a nurse. It’s more like who is she as a person that I can relate with and that breaks down the barrier not necessarily the discipline.

It is likely that no one told Ricardo it is important to find common ground with staff from other disciplines. He simply knows that. When a new social worker arrives who is Japanese, he greets her with the little he knows of her own language. He puts aside his role as ‘nurse’ and hers as ‘social worker’ and instead seeks to get to know her as a person. In another story he told of discovering that the house surgeon played basketball, where upon he said “*Oh hey, so maybe we can have a game sometime, so we did*”. From his experience of being who he already is, Ricardo has come to find the meaning in building relationships with the ‘people’ he works with. It breaks down discipline barriers, opens the way for an ease of communication and helps get things done. Of his relationship with the house surgeon he reflects that if he says “*‘Oh hey can you please change the medication for this person, it just happens very quickly, because you have that relationship to work from*”. Ricardo is a person who enjoys building relationships. It is much more than a competency; it is who he is. As he looks at how other staff struggle to do that with ease, he recognises his gift. He is able to collaborate with people from other disciplines because he knows them and they know him. There is a foundation of trust already established. He has come to appreciate the meaningfulness of working in such a way. Interprofessional practice flourishes because inter-personal relationships, outside the bounds of formal care, have given space for openness to whatever emerges.

Heidegger (1962) talks of the comportment a person brings with them into their everyday encounters. A comportment of openness is characterised by listening with care, being sensitive to others, taking the claims of others seriously, and being ready to be challenged and transformed (Gadamer 1975/2013; Vilhauer 2013). Openness involves keeping the dialectic going. “It is the comportment of openness that leads us… to move beyond the near-sightedness of our own individual perspectives and towards more universal points of view with regards to the subject matter” (Vilhauer 2013, p. 77). Being open and comporting oneself with openness in interprofessional encounters allows a shift in focus from oneself to the patient and others involved in caring for the patient.

Measures of competence can articulate interpersonal skills, but do not capture the spirit of the moment, the chance remark that opens a way, the respectful acknowledgment of ‘other’ that wins their trust. It is the comportment the person brings, shaped by culture, history, life experience, influenced by mood, time and challenge, yet somehow always being-Ricardo that makes the difference. Comportment cannot be measured, but it is always known. Ricardo is the ‘friendly one’ while Japanese social worker is the ‘shy’ one. Who a person is comes with them into their health professional education. Possibilities of who they go on to become may open, or may not. Comportment may not be something that can be taught, yet it can be role modelled. It is not something that can be assessed, but it is always felt. Nor is it something that a person can ‘put on,’ for inauthenticity shines through. ‘How’ each person comports themselves in an interprofessional team always matters yet is likely seldom articulated. It is likely the factor that most influences flourishing.

The last story from Carey, a physiotherapist, reveals the emergent nature of interprofessional practice. It shows how the way toward interprofessional practice was forged through being open, attuned and responsive to the situation that presented itself.

There was a patient who I worked with who had a traumatic brain injury and was in our rehabilitation unit. I remember the first time I met him, he was this little meek guy, who was incontinent, he couldn’t move or express himself and we said his name the wrong way around to start with! He was one of those patients where it really made me realise what other professions do, where my profession fitted in for him. It was in working with this patient that I formed a close working relationship with the psychologist in particular. She was a dominant kind of person to be honest and physio by nature can also attract dominant sort of people into the profession. We started off almost jockeying for position in the schedule to do our own little bits and then we had some discussions and a bit of too-ing and fro-ing about each of our roles and we began to see that we could cross over and deliver each other’s aspects. That’s kind of how it played out. We both sort of began to put our guard down a little bit and opened up our thoughts about our professional roles. I was open to asking “what are you doing?” and “how is that going to work?” which led to the light bulb moment where we both recognised that we could work together and I could do this for her and she could do this for me and that’s going to help the patient. It was actually seeing that our professions and interventions with this patient could cross over that prompted us to work together to identify his specific problems and use each other to deliver a combined treatment programme.

How this situation unfolded brought interprofessional practice into being. There had been no formula to guide how these practitioners should work together with the patient. This journey toward interprofessional practice was one that was open and responsive to the situation, to the patient and to each other. The conversation opened possibilities and shaped how care was to be given over to the patient. It was their encounter through dialogue that created shared understandings and insights.

Gadamer (1975/2013) suggests that genuine dialogue emerges when we engage in conversations that we fall into, which are incomplete, lack structure and don’t follow any rules. The ‘play’ in dialogue happens without effort, “it happens as it were, by itself” (p. 109). Carey illustrates the notion of ‘play’ in dialogue when she talks about the too-ing and fro-ing in the conversation and how the building of understanding came from how the conversation ‘played out’. A topic may come to be more fully understood through the back and forward motion in dialogue and genuinely open conversations (Binding and Tapp 2008). The play of the dialogue in Carey’s and the psychologist’s story just happened and, although it was purposeful, they could not predict how it would end. It appeared to absorb them both, allowing them to open up to one another.

Carey and the psychologist came to share an understanding. Understanding is more than just asserting one’s own view in a dialogue. It is about being transformed, where we are no longer as we were (Gadamer 1975/2013; Laverty 2003). “He [Gadamer] showed that conversation holds possibilities to transform productively not only the understanding of the topic, but also the very being of the participants in the dialogue” (Lawn and Keane 2011, p. 122). This open dialogue with the psychologist provided Carey the opportunity to re-evaluate and consider her position (Lawn and Keane 2011). Carey talks about ‘opening up their thoughts’, a ‘beginning to see’ and ‘recognition’ of how they might work together. This opening up and the mutual coming together of their understandings though dialogue, resulted in a light bulb moment, a broadening of their horizons. For Gadamer, understanding is always the fusion of horizons and understanding is always part of a dialogue and the accommodation of the Other (Gadamer 1975/2013; Lawn and Keane 2011).

In their desire to provide good care, which focussed their minds and moved them closer to true interprofessional practice, they moved beyond mere competent performance of tasks. There was a feeling, a spirit in play, which guided them. Like the cabinetmaker’s apprentice described by Heidegger (1993a, b), whose ability with the wood was more than merely gathering knowledge of what he had to build or how to use his tools, the way they were interacting and relating with one another called for them to attune themselves to the specific needs of the patient. Just as the cabinetmaker is attuned to and responds to the grain in the wood, this practice encounter shows an attunement to the situation, to each other.

There is no measure or assessment for the competence of attunement, of being open to the play, of feeling a sense of ‘rightness’. Such a mode of attuned, responsive practice is born of experience, and a willingness to move beyond familiar ways. While interprofessional competencies signal the direction, the emergence of interprofessional practice in a specific time and place is about the comportment of each player and trust in their sense of knowing. Such practice can be witnessed with a sense of awe, but with no ‘model’ to explain how it came about. It simply unfolded in that specific context, in that particular way. Such is the ontology of interprofessional practice.

## Discussion

Interprofessional competencies, and their associated schema, are representative of an ontic science where “this or that human being” is viewed as an object (Heidegger 2001, p. 222). Such a perspective values ‘knowledge’ as commodity. It can be ascertained, recorded, passed on, imposed and measured. In such a way the ‘knowledge of’ assumes control. Such an approach has been criticized for being reductionist without taking heed of the complex nature of professional practice (Kumagai 2014). It becomes akin to a technology, a means by which the players are programmed to act in pre-determined ways (Heidegger 1993b). From an ontic perspective the players are replaceable. One physiotherapist can be replaced by another. One team of mixed professionals is assumed to be akin to a replacement team of the same mix. In Heidegger’s terms, people become “standing reserve” (1993b, p. 322).

Amanda and her team were certainly guided by ontic knowledge of how to conduct a resuscitation. Ricardo would have known about professional boundaries in relationships with team members. Carey, as a physiotherapist, would have had a firm grasp on the treatment options of her discipline appropriate for a patient with such a diagnosis. We accept that such knowledge is valuable and important. Indeed it does allow ‘any’ practitioner to take on the role allocated. The health system could work no other way. We argue however that in itself such an acceptance of ‘knowledge’ is limiting. It does not open the way for the potentiality lying both within each person, and in the synergy of whatever mix becomes a ‘team’.

An ontological approach takes us as close as we can get to experience itself. Rocha (2016, p. 815) suggests “‘knowledge’ hinted at through and by phenomenological method is less extravagant, and therefore more potent and infectious, than traditional notions of epistemological knowledge”. When a story resonates with our own experience, it does not provide a set of guidelines for us to follow, rather it excites us into our own potential to practice in a more authentic manner. Stories are always about ‘real’ people talking about “specific situations and contexts” (Kumagai 2014, p. 982). We see how the way we are, the cheerful greeting, the helpful hand, or the wise guidance, builds relationships that make a difference. We remember the time when, with another colleague, we tried something new and saw the patient flourish. Our confidence to be and become more fully ourselves is awoken.

We argue that the world is ‘enframed’ (Heidegger 1993b, p. 325) by a technological worldview that is ordered and controlled by a discourse of competencies. Such enframing reveals “the manner in which it allows us, and seemingly compels us, to view the world we live in” (O’Brien 2004, p. 20). Others, in examining the competency movement in health professions, have already called for “more critical debate about the ways and degree to which we use this tool to shape education, regulation and practice,” pointing to the “constraints and conflicts they can impose” (Reeves et al. 2009, p. 453). How then do we move beyond such constraint and tension? O’Brien (2004) interprets Heidegger’s answer to this question as: “We effect this by remaining true and fast to our human voca-tion (vocare), the calling we all have as humans” (p. 38). We are first and foremost ‘human’. Before the concept of ‘competency’ was invented, people worked together in care of the sick. They may not have had professional roles, but they came with a duty of care, a willingness to draw on each other’s expertise, and their own way of being themselves. In such a way they worked together, sometimes flourishing, and other times not.

To embrace an ontological approach to practice and education is to accept that first each person needs to come to know themselves, and then be willing to open themselves in a manner that enables others to know them. It is to strip oneself of the professional mask to be revealed as ‘human’, a person with feelings, ideas, history and hope. Rocha (2016) recognises the fear in such a way: “I do not wish to have the very constitution, the conditions for the possibility of myself, brought into question. The existential core the Socratic command—Know thyself—is terrifying” (p. 10). Perhaps that is why we settle comfortably into the enframing of a system that thinks on our behalf, where ‘I’ can stay safely hidden behind my professional persona.

## Limitations of the study

In keeping with the methodology used in this study, we sought to open questioning and provoke thinking rather than provide answers or solutions to the issues identified. The stories contained within these pages have uncovered meanings of being an interprofessional practitioner and what the allusive and emergent nature of working in a spirit of interprofessional practice might look like. There is, however, so much more to explore and reveal that remains hidden, not yet known. The study pointed toward a way of being interprofessional based on the lived experience of some practitioners, from some health professions, working in some contexts at particular points in time. It did not represent the voices of all health professionals, although the stories and interpretations may resonate with others.

The recruitment methods used drew us toward people who had stories of interprofessional practice to tell, not to people who might be retreating from, or blocking interprofessional practice; people who had tried and failed. We are not to know what other insights they might have shared. We were drawn to focus more on those things which contributed to, and that we felt worked to promote a spirit of interprofessional practice. Those things which worked to constrain interprofessional practice were not dwelled on in any depth. At the start of this journey we did not know what the stories would reveal, therefore deeper more focussed questioning and interpretation related to working in a spirit of interprofessional practice only began to emerge within the later interviews.

## Conclusion

The stories presented in this paper were told as experiences when working together had flourished. The spirit of interprofessional practice was at work. Perhaps the teller (without necessarily saying) recognised they too impacted how things worked. It was their humanness that made the difference; their willingness to be open to others and seize the possibilities of the moment. Openness brings vulnerability and hope. In the moment of reaching out, one has no way of knowing how the other will respond. These three participants, and the others in the study, demonstrated a willingness to explore meaningfulness that arose in relationship. Responding to the needs of ‘this’ patient, working with ‘this’ colleague, meant a unique way forward unfolded beyond pre-determined plans. This is far removed from a system of care that rests on the calculability of human resources where it is assumed that meeting competencies will fit any given situation. Interprofessional competency is an ideal. In contrast sensing a spirit of interprofessional practice is born of lived experience. It is known by a feeling that things are working well (or not). Such a spirit ‘arises’ from the people who work together to ‘do their best’ for the person in their care. In its positive mode, it is known by a sense of satisfaction that things went well, but why that was might be harder to define. Was it the person who led, or the person who came up with an idea, or the enjoyment of each other’s company, or the ease with which everything flowed? Was it all of those and more?

The implications of this study begin with one person from one profession building a relationship with one or more people from another profession. In that relational encounter possibilities are free to emerge. It is always the unique individual that matters. Only the person with openness and willingness to engage will be able to work in a spirit of interprofessional practice. Therefore attention needs to be paid to who the person is and how they are with others. This is elusive—difficult to teach and assess. While we sense when it is being done well, it is hard to pin down. Undergraduate education has possibilities of enabling students to learn from, with and about one another in such a way that they experience the value of engaging in dynamic relationships in the delivery of person centred healthcare. The participant accounts in this article demonstrate that when people become familiar with working in this way interprofessional practice flourishes.

It is our hope that the stories within this article begin to show the rich possibilities that lie in remaining true and fast to our human vocation. When the attuned, responsive authentic self is set free to work with others in a human-to-human manner the rich possibilities of practice reveal themselves in ways that astound and delight.

## Footnotes

1 Recognising that we had perhaps taken our interpretation of this story beyond what was actually said we invited comment from Amanda: “What a lovely interpretation of this story. Yes, it is very much in tune with the lived experience. Knowing something, understanding the algorithm and then doing—putting it all into practice in the moment with a group of diverse people is very different. You have captured that beautifully. It is an openness, it was a moment of interprofessional practice flourishing, a feeling of having come together in a way that created something more—more than each of our competencies as you say, more than anyone of us could have achieved on our own. We became a team fighting for this man's life, a team making a difference”.
